# Common Variants in *NUS1* and *GP2* Genes Contributed to the Risk of Gestational Diabetes Mellitus

**DOI:** 10.3389/fendo.2021.685524

**Published:** 2021-06-29

**Authors:** Tianxiao Zhang, Longrui Zhao, Shujin Wang, Juan Liu, Ying Chang, Louyan Ma, Jia Feng, Yu Niu

**Affiliations:** ^1^ Department of Epidemiology and Biostatistics, School of Public Health, Xi’an Jiaotong University Health Science Center, Xi’an, China; ^2^ Department of Endocrinology and Metabolism, Ninth Hospital of Xi’an, Xi’an, China; ^3^ Department of Forensic Medicine, School of Medicine & Forensics, Xi’an Jiaotong University Health Science Center, Xi’an, China; ^4^ Department of Obstetrics, Northwest Women and Children’s Hospital, Xi’an, China; ^5^ Department of Pharmacy, Northwest Women and Children’s Hospital, Xi’an, China; ^6^ Department of General Practice, Ninth Hospital of Xi’an, Xi’an, China

**Keywords:** gestational diabetes mellitus, single nucleotide polymorphism, case-control study, *NUS1*, *GP2*

## Abstract

**Background:**

Recently, *NUS1* and *GP2* genes were reported to be associated with the risk of type 2 diabetes (T2D) in a Japanese population. Given the sharing of pathogenic contribution from genetic factors between T2D and gestational diabetes mellitus (GDM), we conducted the study to systematically examine the relationship of *NUS1* and *GP2* genes with the susceptibility to GDM in Chinese Han population.

**Methods:**

A total of 4,250 subjects comprised of 1,282 patients with GDM and 2,968 controls were recruited, and 20 tag single nucleotide polymorphisms (SNPs) (10 from *NUS1* and 10 from *GP2*) were selected for genotyping. Association analyses were conducted for GDM and its related biomedical indexes including fasting glucose and HbA1c levels.

**Results:**

Two SNPs, rs80196932 from *NUS1* (*P*=2.93×10^-5^) and rs117267808 from *GP2* (*P*=5.68×10^-5^), were identified to be significantly associated with the risk of GDM. Additionally, SNP rs80196932 was significantly associated with HbA1c level in both patients with GDM (*P*=0.0009) and controls (*P*=0.0003), while SNP rs117267808 was significantly associated with fasting glucose level in both patients with GDM (*P*=0.0008) and controls (*P*=0.0007). Serum levels of protein NUS1 and GP2 were measured for the study subjects, and significant differences were identified among groups with different genotypes of SNP rs80196932 and rs117267808, respectively.

**Conclusions:**

Our findings indicate that NUS1 and GP2 genes contribute to the risk of GDM, which would help to offer the potential to improve our understanding of the etiology of GDM and, in turn, could facilitate the development of novel medicines and treatments for GDM.

## Introduction

Gestational diabetes mellitus (GDM) is diabetes diagnosed for the first time during pregnancy (1–3). It is the most common metabolic disorder in pregnant women (1). The reported prevalence of GDM varies from 1% to >30% due to a lack of consensus in diagnostic criteria for GDM (4). The prevalence of GDM is highest in the Middle East and North Africa (median, 15.2%) and lowest in Europe (median, 6.1%) (4–6). Similar to type 2 diabetes (T2D), insulin is the primary medical treatment for GDM if lifestyle intervention is not effective in controlling glucose (4). The diagnosis of GDM for most women is potentially associated with anxiety related to maternal and fetal health (7). Depression has also been reported to be more frequent in women with GDM than in pregnant women without GDM (8–10). Additionally, GDM has been reported to be associated with an increased risk of later maternal diabetes and cardiovascular disease (11).

Elucidating the underlying pathological mechanisms of GDM could facilitate the development of novel treatments and disease prevention for GDM. Similar to T2D, GDM is considered a complex disorder with contributions from multiple genetic and environmental factors (12, 13). Up to now, two genome-wide association (GWA) studies have been published, and multiple loci have been reported to be associated with susceptibility to GDM including *IGF2BP2*, *CDKAL1*, *SLITRK6*, *NUMBL*, *LTBP4*, and *SNRPGP16* (14, 15), and most of the reported significant loci were identified in previous GWA studies for T2D.

A recent GWA study reported genetic polymorphisms associated with the risk of T2D in two novel loci, the *NUS1* and *GP2* genes, in a Japanese population (16). Suzuki et al. identified that the T allele of SNP rs80196932 in *NUS1* is associated with a reduced risk of T2D. Additionally, they found that the A allele of single nucleotide polymorphism (SNP) rs117267808 in *GP2* is associated with an increased risk of T2D. *NUS1* gene encodes a transmembrane domain protein which is a subunit of cis-prenyltransferase (17). *GP2* gene encodes a protein named glycoprotein 2 which is an integral membrane protein (18). This protein binds pathogens such as bacteria and therefore plays a significant role in the immune response (18). Previous evidence has indicated that both T2D and GDM might share a common genetic basis (19–21). Given the sharing of genetic contribution between T2D and GDM in their pathogenesis, we hypothesized that *NUS1* and *GP2* may also contribute to the risk of GDM. To examine this hypothesis, we performed a case-control study to evaluate the potential association between *NUS1* and *GP2* and the risk of GDM in a sample of women with Chinese Han ancestry. The aim of this study was to systematically examine the relationship of the common DNA variants in *NUS1* and *GP2* with the susceptibility for GDM. This study will illuminate the pathological mechanisms involved in GDM.

## Methods

### Study Subjects

We conducted a case-control study to evaluate genetic association. Both patients with GDM and healthy controls were enrolled from the Ninth Hospital of Xi’an and Northwest Women and Children’s Hospital from April 2015 to June 2019. All study subjects were unrelated women at 24 weeks to 28 weeks of gestation. Individuals with metabolism-related disorders, including hypertension, diabetes, previous polycystic ovary syndrome, autoimmune disease, and preeclampsia, pregnancy-induced hypertension, were excluded. Additionally, women with alcohol abuse or multiple gestation were not included in this study. Controls were healthy pregnant women without any maternal or fetal disorders. The diagnosis of GDM was made according to guidelines proposed by the International Association of the Diabetes and Pregnancy Study Groups (IADPSG) in 2010. Fasting whole-blood samples were collected for analyzing of basic biochemical data including fasting glucose and HbA1c profiles. Another 5 ml of maternal venous blood was drawn from each enrolled case upon admission for genotyping experiments. Demographic and clinical variables including age, prepregnancy BMI, and family history of diabetes mellitus were collected by questionnaire. This study was performed in accordance with the ethical guidelines of the Declaration of Helsinki (version 2002) and was approved by the Ethics Committee of the Ninth Hospital of Xi’an. Written consent forms were collected from all study subjects.

### SNP Selection and Experimental Methods

To investigate the potential contributions of *NUS1* and *GP2* genes to GDM risk, tag SNPs of the two genes were selected for genotyping. We first extracted 88 SNPs located within the genetic regions of *NUS1* and *GP2* with minor allele frequency (MAF) ≥0.05 based on Han Chinese data from the 1000 genome project (https://www.internationalgenome.org/). Then, tag SNPs were selected using r^2^>0.8 as criteria. Finally, 20 tag SNPs (10 for *NUS1* and 10 for *GP2*) were selected and genotyped.

We extracted the genomic DNA from the peripheral blood samples of the study subjects using a DNA extraction kit provided by Tiangen Biotech Co. (Tiangen Biotech Co. Ltd, Beijing, China). SNP genotyping was implemented with the Sequenom MassARRAY platform (Sequenom, San Diego, CA, USA). Raw genotyping data were processed by Sequenom Typer 4.0 software. To evaluate the accuracy of the genotyping experiment, 5% of the samples were randomly selected for replication. A 100% concordance rate was obtained for the replication experiment results. In addition, serum level of protein NUS1 and GP2 in the study subjects were measured using enzyme-linked immunosorbent assay (ELISA) kits manufactured by Westtang Biotech Inc.(Shanghai, China).

### Statistical Analysis

Demographic and characteristic information of study subjects were compared between patients with GDM and controls by Student’s t*-*test or χ^2^ test. Hardy-Weinberg equilibrium (HWE) was tested in controls for all SNPs using χ^2^ test. Single-marker-based association analyses were conducted in allelic and genotypic groups. Logistic models were fitted for significant hits obtained from single-marker-based association analyses, and age and prepregnancy BMI were included as covariates. Linkage disequilibrium (LD) blocks were constructed using genetic data from selected SNPs. Haplotype-based association analysis was conducted within each LD block. In addition to risk of GDM, fasting glucose and HbA1c levels were also analyzed as phenotypes using linear models stratified by the disease status of our study subjects. We have performed ANOVA to investigate the distributions of serum level of protein NUS1 and GP2 in groups with different genotypes of SNP rs80196932 and rs117267808. Plink was utilized for genetic association analysis (22). Haploview was utilized for visualization of the LD blocks (23). Bonferroni corrections were applied to multiple comparisons and 0.0025 was chosen as threshold of *P* values for single-marker-based association analyses.

### Bioinformatics Analysis

To further investigate the potential functional consequences of significant SNPs, expression quantitative trait loci (eQTL) data from the GTEx database were extracted (https://www.gtexportal.org/home/) (24). eQTL data from 47 types of human tissue were extracted and examined.

## Results

### Demographic and Clinical Characteristics of the Study Subjects

A total of 4,250 study subjects comprised of 1,282 patients with GDM and 2,968 controls were recruited (**Table 1**). No significant difference was identified for age and prepregnancy BMI between patients with GDM and controls. Significant differences were identified for parity (*P*=0.0069), abnormal pregnancy (*P*=0.0030), and family history of diabetes mellitus (*P*=0.0038) between patients with GDM and controls.

**Table 1:** Demographic and clinical characteristics of the study subjects.

Variables	Cases (N=1,282)	Controls (N=2,968)	Statistics	*P*
Age, years	30.6 ± 3.7	30.4 ± 4.2	t = 1.35	0.1784
Prepregnancy BMI, kg/m^2^	22.3 ± 1.4	22.2 ± 1.4	t = 1.74	0.0829
Fasting Glucose, mmol/l	6.9 ± 0.9	4.6 ± 0.2	t = 86.63	< 2.2×10^-16^
HbA1c, mmol/mol	7.3 ± 0.5	5.2 ± 0.5	t = 122.68	< 2.2×10^-16^
Parity (%)				
* Nulliparae*	1,159 (90)	2,757 (93)		
* Multiparae*	123 (10)	211 (7)	χ^2^ = 7.30	0.0069
Abnormal Pregnancy (%)				
* Yes*	80 (6)	121 (4)		
* No*	1,202 (94)	2,847 (96)	χ^2^ = 8.83	0.0030
Family History of Diabetes Mellitus (%)				
* Yes*	173 (13)	308 (10)		
* No*	1,109 (87)	2,660 (90)	χ^2^ = 8.36	0.0038

Age, prepregnancy BMI, fasting glucose and HbA1c level are presented by mean ± sd.

### Significant SNPs Contributing to the Risk of GDM

No significant results of HWE tests were identified for the 20 SNPs in controls (**Supplemental Table S1**). We identified two significant SNPs, SNP rs80196932 from *NUS1* and SNP rs117267808 from *GP2*, associated with the risk of GDM (**Table 2** and **Supplemental Table S2**). The C allele of rs80196932 was significantly associated with a reduced risk of GDM [OR (95%CI)=0.74 (0.64-0.85), χ^2^ = 17.47, *P*=2.93×10^-5^]. On the other hand, the A allele of SNP rs117267808 was significantly associated with an increased risk of GDM [OR (95%CI)=1.41 (1.19-1.67), χ^2^ = 16.21, *P*=5.68×10^-5^]. Dose-response relationships were observed for both significant SNPs in the genotypic analyses. These significant signals remained after adjusting for age and prepregnancy BMI using logistic models (**Table 2** and **Supplemental Table S3**).

**Table 2:** Significant results for single marker based association analyses.

CHR	SNP	A1	Genotypic Analysis						Allelic Analysis						*P
			Genotypes	Patients	Controls	OR[95% CI]	χ^2^	*P*	Alleles	Patients	Controls	OR[95% CI]	χ^2^	*P*	
6	rs80196932	C	CC	15	65	0.50[0.28-0.87]									
			CT	262	748	0.75[0.64-0.88]			C	292	878	0.74[0.64-0.85]			
			TT	1,005	2,155	ref	17.53	0.0002	T	2,272	5,058	ref	17.47	2.93×10^-5^	2.79×10^-5^
16	rs117267808	A	AA	9	9	2.44[0.97-6.18]									
			AG	218	380	1.40[1.17-1.68]			A	236	398	1.41[1.19-1.67]			
			GG	1,055	2,579	ref	16.81	0.0002	G	2,328	5,538	ref	16.21	5.68×10^-5^	6.20×10^-5^

CHR, chromosome; A1, tested allele (minor allele); OR [95%CI], odds ratio with 95% confidence interval.

Threshold for P-Value was 0.05/20 = 0.0025.

*P values after being adjusted for age and prepregnancy BMI using logistic models.

### Significant Haplotypes Associated With the Risk of GDM

6 LD blocks were constructed using genetic data of the selected SNPs (**Supplemental Figure S1**). Significant haplotypes were identified for both *NUS1* and *GP2* (**Supplemental Table S4**). In *NUS1*, a two-SNP block including rs80196932 and rs9767451 was identified to be significantly related to the disease status of GDM (χ^2^ = 30.30, *P*=2.64×10^-7^). In *GP2*, a three-SNP block including rs117267808, rs141536185, and rs4430753 was identified to be significantly related to the disease status of GDM (χ^2^ = 29.33, *P*=1.91×10^-6^). Not surprisingly, both significant LD blocks contained the significant hits found in single marker-based analyses.

### Both rs80196932 and rs117267808 Are Significantly Associated With Diabetes-Related Biomedical Indexes

In addition to the risk of GDM, we also investigated the association between the significant genetic markers and diabetes-related biomedical indexes including fasting glucose and HbA1c levels (**Table 3**). Age and prepregnancy BMI were included in the linear model as covariates. This analysis was conducted separately for patients with GDM and controls, and the results from both groups agreed with each other. SNP rs80196932 was significantly associated with the HbA1c level. However, this SNP was not associated with the fasting glucose level. The C allele of rs80196932 was significantly related with reduced HbA1c level in both patients with GDM (t-statistic=-3.34, *P*=0.0009) and controls (t-statistic=-3.66, *P*=0.0003). On the other hand, SNP rs117267808 was significantly associated with the fasting glucose level but not the HbA1c level. The A allele of rs117267808 was significantly related with increased fasting glucose level in both patients with GDM (t-statistic=3.36, *P*=0.0008) and controls (t-statistic=3.38, *P*=0.0007).

**Table 3:** Associations between significant SNPs and GDM related biomedical indexes stratified by disease status.

Status	Biomedical Indexes (Mean ± SD)	SNP	Gene	*β*	*t*-statistics	*P*
Patients with GDM (N=1,282)	Fasting Glucose (6.9 ± 0.9)	rs80196932	*NUS1*	0.03	0.50	0.6182
		rs117267808	*GP2*	0.22	3.36	0.0008
	HbA1c (7.3 ± 0.5)	rs80196932	*NUS1*	-0.11	-3.34	0.0009
		rs117267808	*GP2*	0.02	0.58	0.5630
Controls (N=2,968)	Fasting Glucose (4.6 ± 0.2)	rs80196932	*NUS1*	-0.01	-0.87	0.3820
		rs117267808	*GP2*	0.04	3.38	0.0007
	HbA1c (5.2 ± 0.5)	rs80196932	*NUS1*	-0.07	-3.66	0.0003
		rs117267808	*GP2*	-0.02	-0.78	0.4375

### eQTL Signals Identified for SNP rs80196932 in NUS1

**Figure 1:**
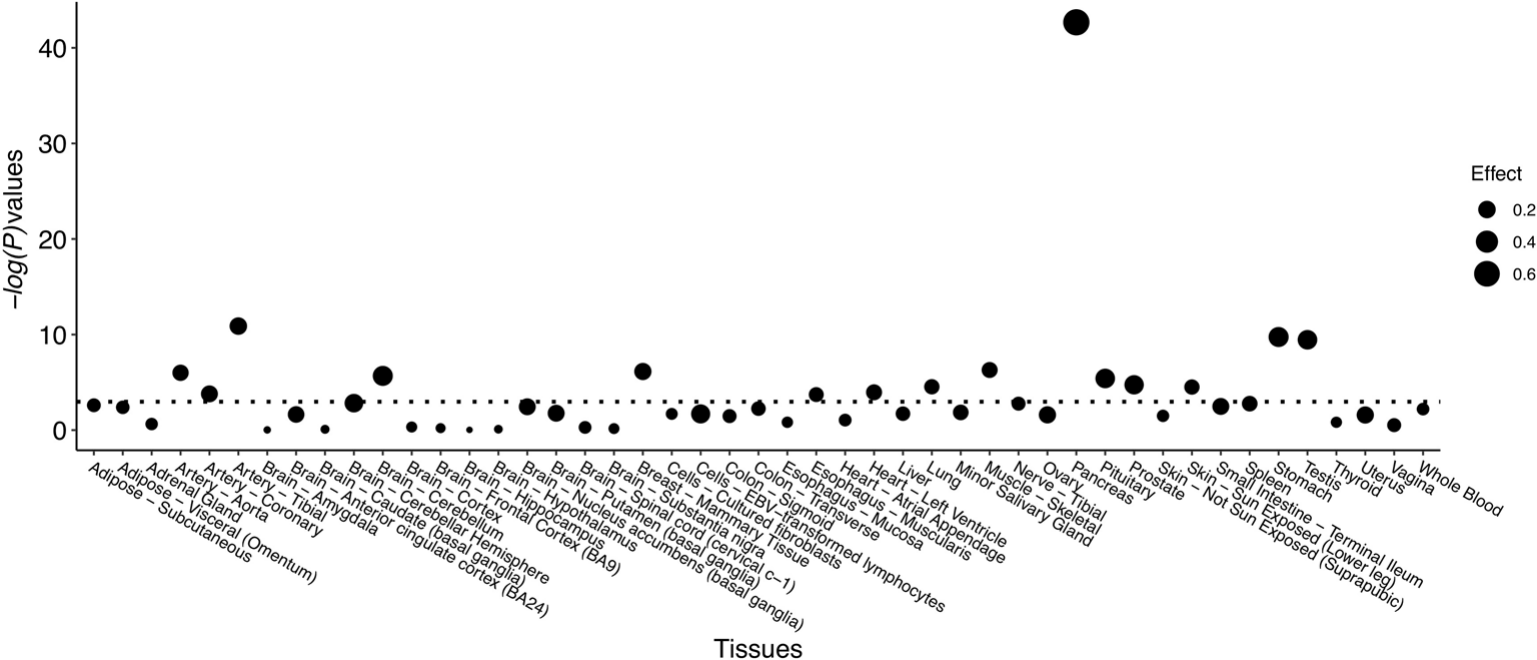
eQTL signals identified for SNP rs80196932 on gene *NUS1* from multiple types of human tissues. Threshold of -log(*P*-value) is indicated by dotted line.

Significant eQTL signals were discovered for SNP rs80196932 in the *NUS1* gene from multiple types of human tissues (**Figure 1** and **Supplemental Table S5**). The most significant eQTL signal was obtained from pancreatic tissue, which is the target tissue of GDM. The C allele of rs80196932 was significantly related with increased expression of *NUS1* gene in pancreatic tissue (**Supplemental Figure S2**). No significant eQTL signals were found for SNP rs117267808 on the *GP2* gene (**Supplemental Table S6**).

### Association of Serum NUS1 and GP2 Level With Significant SNPs

**Figure 2:**
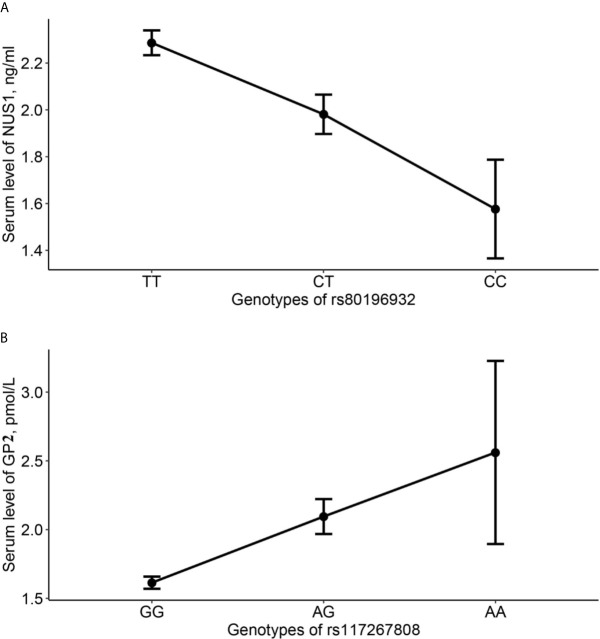
Average serum level of NUS1 and GP2 in different genotype groups of SNP rs80196932 and rs117267808. **(A)** Serum level of NUS1 in different genotype groups of SNP rs80196932. **(B)** Serum level of GP2 in different genotype groups of SNP rs117267808. 95% confidence intervals were indicated by the error bars.

Genotypes of SNP rs80196932 and rs117267808 are significantly associated with serum level of NUS1 and GP2, respectively. The C allele of SNP rs80196932 was significantly related with decreased serum level of protein NUS1 (**Figure 2A**). The A allele of SNP rs117267808 was significantly related with decreased serum level of protein GP2 (**Figure 2B**). Further stratification analysis indicated that these trends are in the same direction in both GDM cases and controls (**Supplemental Table S7**).

## Discussion

In this study we identified genetic polymorphisms of the *NUS1* and *GP2* genes contributing to the risk of GDM in a sample of Chinese women with Han ethnic group ancestry. Although both *NUS1* and *GP2* have been reported to contribute to the risk of T2D in a recent GWA study in a Japanese population (16), our study is the first to link the two loci to GDM in samples with Chinese Han ancestry. The direction of effect for both SNPs on GDM discovered in the present study was consistent with that reported in the recent T2D GWA study, although the effect sizes of both SNPs were much smaller in the recent GWA study.

SNP rs80196932 is located in the 5’ untranslated region of *NUS1*. According to the RegulomeDB database, it located in an evolutionarily conserved region which overlaps with DNase I–hypersensitive sites and active transcriptional start sites (25). Additionally, in the bioinformatics analysis, we identified multiple significant eQTL signals for this SNP in the *NUS1* gene in various types of human tissues including the pancreas. Therefore, this SNP may be more than a surrogate and may be a susceptible DNA variant with true functional consequences.

The eQTL evidence showed that the C allele of SNP rs80196932 was significantly related with increased expression level of *NUS1* gene in human tissue of pancreas. However, at the protein level, we have identified the C allele of SNP rs80196932 was significantly associated with decreased serum level of protein NUS1 in the study subjects. This discordant finding could be due to multiple reasons. First of all, the eQTL signals were extracted from human tissue of pancreas while the protein level of NUS1 was measured in serum. This spatial difference might affect the gene expression and translation. In addition, complex mechanisms post-transcriptional regulations, such as phosphorylation and ubiquitination, might complicate the relationship between gene expression level and protein level.

Although it is out of the scope of the current study to examine the pathological mechanisms of *NUS1* in GDM, we can still obtain some clues by integrating evidence from the present study. According to our findings, the C allele of SNP rs80196932 was associated with a lower risk of GDM and with higher expression of *NUS1*. Previous studies have indicated that *NUS1* is essential for protein glycosylation and intracellular cholesterol trafficking (26), and another animal model-based study showed that mouse embryonic fibroblasts with conditional knockdown of *Nus1* accumulated free cholesterol (17). On the other hand, plasma cholesterol levels have long been considered to be associated with the risk of diabetes (27, 28). In this sense, we believe that the C allele of SNP rs80196932 reduces the risk of GDM by increasing the expression level *NUS1* and in turn reducing free cholesterol levels. More studies are still needed to unravel the underlying pathological mechanisms of *NUS1* and GDM.

SNP rs117267808 is an intronic DNA variant of *GP2* gene. Although we have identified significant relationship between genotypes of rs117267808 and serum level of protein GP2, no significant eQTL signals for rs117267808 on *GP2* were found. Therefore, the functional consequences of this DNA variant on the *GP2* gene are still not clear. It is probably that rs117267808 is a surrogate of some underlying DNA variants with true effects. A recent study showed that GP2 is a marker for human pancreatic progenitors cells and it secrete digestive enzymes and confers plasticity to convert progenitors into insulin-producing beta cells (29). However, we were not able to illuminate the mechanisms underlying GDM pathology and *GP2* in the present study.

Our findings from association analysis of diabetes-related biomedical indexes indicated that although *NUS1* and *GP2* both contribute to the risk of GDM, they might be involved in different mechanisms of GDM. SNP rs80196932 from *NUS1* and SNP rs117267808 from *GP2* were associated with different biomedical indexes. It seems that *NUS1* was related to HbA1c, while *GP2* was related to fasting glucose. This difference can be partially explained by the difference in the functions of the two genes. Although it is insufficient to draw solid conclusions only from SNP results (30–32), the protein product of *GP2* has been reported to be causally related to insulin-producing beta cells and therefore could have direct effects on the fasting glucose level (29). On the other hand, *NUS1* is related to the protein glycosylation and intracellular cholesterol trafficking. A recent study demonstrated correlation between HbA1c and serum lipid profiles in T2D patients (33). Therefore, *NUS1* might be associated with HbA1c levels through its effect on cholesterol trafficking.

Our study has several limitations. Population stratification is a main confounding factor for population based gene association mapping studies. As a study based on a couple of candidate SNPs it is impossible for us to address this issue using standard methods such as principal component analysis. However, we tried to implement additional inclusion criteria to at least partially address this potential issue. In the subject enrollment process, we only recruited native individuals with no immigration history in last three generations. In this sense, we could, at least partly, restrict the genetic heterogeneity of the study subjects. Another potential limitation is that the eQTL evidence of the present study was extracted from a publicly available database which was based on individuals with no GDM. However, the expression levels for genes could be different in controls and patients with GDM. Thus, caution is advised for interpreting the results of eQTL signals. Last but not least, as this study only genotyped several candidate SNPs, we might miss the opportunity to discover novel susceptible variants of GDM.

In summary, we reported novel association signals between genetic polymorphisms of the *NUS1* and *GP2* genes and GDM risk. Associations with diabetes-related biomedical indexes were also identified. Our findings shed light on the roles of *NUS1* and *GP2* in pathogenic mechanisms of GDM, which will be further elucidated in future investigation.

## Data Availability Statement

The datasets generated during the current study are not publicly available due to local legislation, but are partially available from the corresponding author on reasonable request (Email: yuniuxjtu@163.com).

## Ethics Statement

The studies involving human participants were reviewed and approved by Ethics Committee of the Ninth Hospital of Xi’an. The patients/participants provided their written informed consent to participate in this study.

## Author Contributions

Authors YN and TZ conceived and designed the study. TZ and LZ carried out candidate SNPs selection and statistical analyses. SW, JL, YC, LM, JF, and YN conducted subject screening. TZ, LZ, and YN contributed to the collection and preparation of control DNA samples. TZ and YN drafted the first version of manuscript together. All authors contributed to the article and approved the submitted version.

## Funding

This research was totally supported by National Natural Science Foundation of China (No. 81800711), Shaanxi Province Health Research Funded Project (No. 2018D004). The funding sources had no role in the design of this study, the collection, analysis and interpretation of data, the writing of the report, or the decision to submit the paper for publication.

## Conflict of Interest

The authors declare that the research was conducted in the absence of any commercial or financial relationships that could be construed as a potential conflict of interest.

## References

[1] Chiefari E Arcidiacono B Foti D Brunetti A. Gestational Diabetes Mellitus: An Updated Overview. J Endocrinol Invest (2017) 40(9):899–909. 10.1007/s40618-016-0607-5 28283913

[2] Johns EC Denison FC Norman JE Reynolds RM. Gestational Diabetes Mellitus: Mechanisms, Treatment, and Complications. Trends Endocrinol Metab (2018) 29(11):743–54. 10.1016/j.tem.2018.09.004 30297319

[3] McIntyre HD Catalano P Zhang C Desoye G Mathiesen ER Damm P. Gestational Diabetes Mellitus. Nat Rev Dis Primers (2019) 5(1):47. 10.1016/j.ecl.2019.05.001 31296866

[4] Dickens LT Thomas CC. Updates in Gestational Diabetes Prevalence, Treatment, and Health Policy. Curr Diabetes Rep (2019) 19(6):33. 10.1007/s11892-019-1147-0 31073850

[5] Lee KW Ching SM Ramachandran V Yee A Hoo FK Chia YC. Prevalence and Risk Factors of Gestational Diabetes Mellitus in Asia: A Systematic Review and Meta-Analysis. BMC Pregnancy Childbirth (2018) 18(1):494. 10.1186/s12884-018-2131-4 30547769 PMC6295048

[6] Eades CE Cameron DM Evans JMM. Prevalence of Gestational Diabetes Mellitus in Europe: A Meta-Analysis. Diabetes Res Clin Pract (2017) 129:173–81. 10.1016/j.diabres.2017.03.030 28531829

[7] Daniells S Grenyer BF Davis WS Coleman KJ Burgess JA Moses RG. Gestational Diabetes Mellitus: Is a Diagnosis Associated With an Increase in Maternal Anxiety and Stress in the Short and Intermediate Term? Diabetes Care (2003) 26(2):385–9. 10.2337/diacare.26.2.385 12547867

[8] Hinkle SN Buck Louis GM Rawal S Zhu Y Albert PS Zhang C. A Longitudinal Study of Depression and Gestational Diabetes in Pregnancy and the Postpartum Period. Diabetologia (2016) 59(12):2594–602. 10.1007/s00125-016-4086-1 PMC5101167 27640810

[9] Byrn M Penckofer S. The Relationship Between Gestational Diabetes and Antenatal Depression. J Obstet Gynecol Neonatal Nurs (2015) 44(2):246–55. 10.1111/1552-6909.12554 25712378

[10] Nicklas JM Miller LJ Zera CA Davis RB Levkoff SE Seely EW. Factors Associated With Depressive Symptoms in the Early Postpartum Period Among Women With Recent Gestational Diabetes Mellitus. Matern Child Health J (2013) 17(9):1665–72. 10.1007/s10995-012-1180-y PMC3580121 23124798

[11] Kramer CK Campbell S Retnakaran R. Gestational Diabetes and the Risk of Cardiovascular Disease in Women: A Systematic Review and Meta-Analysis. Diabetologia (2019) 62(6):905–14. 10.1007/s00125-019-4840-2 30843102

[12] Rosik J Szostak B Machaj F Pawlik A. The Role of Genetics and Epigenetics in the Pathogenesis of Gestational Diabetes Mellitus. Ann Hum Genet (2020) 84(2):114–24. 10.1111/ahg.12356 31571208

[13] Lowe WL Jr Scholtens DM Sandler V Hayes MG. Genetics of Gestational Diabetes Mellitus and Maternal Metabolism. Curr Diabetes Rep (2016) 16(2):15. 10.1007/s11892-015-0709-z 26803651

[14] Wu NN Zhao D Ma W Lang JN Liu SM Fu Y. A Genome-Wide Association Study of Gestational Diabetes Mellitus in Chinese Women. J Matern Fetal Neonatal Med (2019) 15:1–8. 10.1080/14767058.2019.1640205 31269844

[15] Ding M Chavarro J Olsen S Lin Y Ley SH Bao W. Genetic Variants of Gestational Diabetes Mellitus: A Study of 112 SNPs Among 8722 Women in Two Independent Populations. Diabetologia (2018) 61(8):1758–68. 10.1007/s00125-018-4637-8 PMC6701842 29947923

[16] Suzuki K Akiyama M Ishigaki K Kanai M Hosoe J Shojima N. Identification of 28 New Susceptibility Loci for Type 2 Diabetes in the Japanese Population. Nat Genet (2019) 51(3):379–86. 10.1038/s41588-018-0332-4 30718926

[17] Park EJ Grabińska KA Guan Z Stránecký V Hartmannová H Hodaňová K. Mutation of Nogo-B Receptor, a Subunit of Cis-Prenyltransferase, Causes a Congenital Disorder of Glycosylation. Cell Metab (2014) 20(3):448–57. 10.1016/j.cmet.2014.06.016 PMC4161961 25066056

[18] Hase K Kawano K Nochi T Pontes GS Fukuda S Ebisawa M. Uptake Through Glycoprotein 2 of FimH(+) Bacteria by M Cells Initiates Mucosal Immune Response. Nature (2009) 462(7270):226–30. 10.1038/nature08529 19907495

[19] Kawai VK Levinson RT Adefurin A Kurnik D Collier SP Conway D. A Genetic Risk Score That Includes Common Type 2 Diabetes Risk Variants Is Associated With Gestational Diabetes. Clin Endocrinol (Oxf) (2017) 87(2):149–55. 10.1111/cen.13356 PMC5533106 28429832

[20] Cho YM Kim TH Lim S Choi SH Shin HD Lee HK. Type 2 Diabetes-Associated Genetic Variants Discovered in the Recent Genome-Wide Association Studies are Related to Gestational Diabetes Mellitus in the Korean Population. Diabetologia (2009) 52(2):253–61. 10.1007/s00125-008-1196-4 19002430

[21] Robitaille J Grant AM. The Genetics of Gestational Diabetes Mellitus: Evidence for Relationship With Type 2 Diabetes Mellitus. Genet Med (2008) 10(4):240–50. 10.1097/GIM.0b013e31816b8710 18414206

[22] Purcell S Neale B Todd-Brown K Thomas L Ferreira MA Bender D. PLINK: A Tool Set for Whole-Genome Association and Population-Based Linkage Analyses. Am J Hum Genet (2007) 81(3):559–75. 10.1086/519795 PMC1950838 17701901

[23] Barrett JC. Haploview: Visualization and Analysis of SNP Genotype Data. Cold Spring Harb Protoc (2009) 2009(10):pdb.ip71. 10.1101/pdb.ip71 20147036

[24] GTEx Consortium. The Genotype-Tissue Expression (GTEx) Project. Nat Genet (2013) 45(6):580–5. 10.1038/ng.2653 PMC4010069 23715323

[25] Dong S Boyle AP. Predicting Functional Variants in Enhancer and Promoter Elements Using RegulomeDB. Hum Mutat (2019) 40(9):1292–8. 10.1002/humu.23791 PMC6744346 31228310

[26] Harrison KD Miao RQ Fernandez-Hernándo C Suárez Y Dávalos A Sessa WC. Nogo-B Receptor Stabilizes Niemann-Pick Type C2 Protein and Regulates Intracellular Cholesterol Trafficking. Cell Metab (2009) 10(3):208–18. 10.1016/j.cmet.2009.07.003 PMC2739452 19723497

[27] Perego C Da Dalt L Pirillo A Galli A Catapano AL Norata GD. Cholesterol Metabolism, Pancreatic β-Cell Function and Diabetes. Biochim Biophys Acta Mol Basis Dis (2019) 1865(9):2149–56. 10.1016/j.bbadis.2019.04.012 31029825

[28] Lotta LA Sharp SJ Burgess S Perry JRB Stewart ID Willems SM. Association Between Low-Density Lipoprotein Cholesterol-Lowering Genetic Variants and Risk of Type 2 Diabetes: A Meta-Analysis. JAMA (2016) 316(13):1383–91. 10.1001/jama.2016.14568 PMC5386134 27701660

[29] Cogger KF Sinha A Sarangi F McGaugh EC Saunders D Dorrell C. Glycoprotein 2 Is a Specific Cell Surface Marker of Human Pancreatic Progenitors. Nat Commun (2017) 8(1):331. 10.1038/s41467-017-00561-0 28835709 PMC5569081

[30] Guan F Zhang T Han W Zhu L Ni T Lin H. Relationship of SNAP25 Variants With Schizophrenia and Antipsychotic-Induced Weight Change in Large-Scale Schizophrenia Patients. Schizophr Res (2020) 215:250–5. 10.1016/j.schres.2019.09.015 31653583

[31] Zhang B Chang L Lan X Asif N Guan F Fu D. Genome-Wide Definition of Selective Sweeps Reveals Molecular Evidence of Trait-Driven Domestication Among Elite Goat (Capra Species) Breeds for the Production of Dairy, Cashmere, and Meat. Gigascience (2018) 7(12):giy105. 10.1093/gigascience PMC6287099 30165633

[32] Guan F Ni T Han W Lin H Zhang B Chen G. Evaluation of the Relationships of the WBP1L Gene With Schizophrenia and the General Psychopathology Scale Based on a Case-Control Study. Am J Med Genet B Neuropsychiatr Genet (2020) 183:164–71. 10.1002/ajmg.b.32773 31840934

[33] Hussain A Ali I Ijaz M Rahim A. Correlation Between Hemoglobin A1c and Serum Lipid Profile in Afghani Patients With Type 2 Diabetes: Hemoglobin A1c Prognosticates Dyslipidemia. Ther Adv Endocrinol Metab (2017) 8(4):51–7. 10.1177/2042018817692296 PMC5415005 28507727

