# Supplementary material for: Common Variants in NUS1 and GP2 Genes Contributed to the Risk of Gestational Diabetes Mellitus
Source: Front Endocrinol (Lausanne). 2021 Jun 29;12:685524. doi: 10.3389/fendo.2021.685524 (PMC8315097; doi:10.3389/fendo.2021.685524)
Supplement: Supplementary file 1 [file DataSheet_1.docx]

**Supplemental Table S1.** Genetic information of the 20 selected SNPs.

| CHR | POS | SNP | Gene | Function | A1 | A2 | MAF | HWE |
| --- | --- | --- | --- | --- | --- | --- | --- | --- |
| 6 | 117675468 | rs80196932 | *NUS1* | untranslated-5 | C | T | 0.14 | 1.00 |
| 6 | 117675655 | rs9767451 | *NUS1* | untranslated-5 | T | C | 0.18 | 0.25 |
| 6 | 117676293 | rs72967259 | *NUS1* | intron | T | C | 0.10 | 0.24 |
| 6 | 117685173 | rs77885036 | *NUS1* | intron | T | A | 0.10 | 0.14 |
| 6 | 117686129 | rs11153693 | *NUS1* | intron | C | T | 0.38 | 0.70 |
| 6 | 117686329 | rs74296487 | *NUS1* | intron | G | A | 0.07 | 0.25 |
| 6 | 117695136 | rs111583609 | *NUS1* | intron | A | G | 0.28 | 0.23 |
| 6 | 117695611 | rs6899715 | *NUS1* | intron | A | G | 0.34 | 0.29 |
| 6 | 117699138 | rs78845922 | *NUS1* | intron | T | C | 0.13 | 0.29 |
| 6 | 117702153 | rs1541317 | *NUS1* | intron | G | T | 0.28 | 0.50 |
| 16 | 20309986 | rs12922283 | *GP2* | untranslated-3 | C | G | 0.17 | 0.36 |
| 16 | 20310838 | rs12596974 | *GP2* | untranslated-3 | C | A | 0.28 | 0.53 |
| 16 | 20311846 | rs117267808 | *GP2* | intron | A | G | 0.07 | 0.24 |
| 16 | 20315755 | rs141536185 | *GP2* | intron | A | G | 0.06 | 0.87 |
| 16 | 20320878 | rs4430753 | *GP2* | intron | A | G | 0.20 | 0.68 |
| 16 | 20321195 | rs7185180 | *GP2* | intron | C | T | 0.08 | 0.60 |
| 16 | 20323357 | rs4411505 | *GP2* | intron | C | T | 0.13 | 0.43 |
| 16 | 20323485 | rs4420538 | *GP2* | intron | T | A | 0.13 | 0.43 |
| 16 | 20324935 | rs11074451 | *GP2* | intron | G | A | 0.09 | 0.47 |
| 16 | 20326883 | rs4643331 | *GP2* | intron | T | C | 0.10 | 0.76 |

CHR: chromosome; POS: position; A1: tested allele (minor allele); A2: major allele; MAF: minor allele frequency; HWE: *P* values for Hardy-Weinberg equilibrium tests conducted in controls.

**Supplemental Table S2.** Full results for single marker based association analysis.

| CHR | SNP | A1 | A2 | TEST | AFF | UNAFF | χ^2^ | *P* |
| --- | --- | --- | --- | --- | --- | --- | --- | --- |
| 6 | rs80196932 | C | T | GENO | 15/262/1005 | 65/748/2155 | 17.53 | 0.0002 |
|  |  |  |  | ALLELIC | 292/2272 | 878/5058 | 17.47 | 2.93×10^-5^ |
| 6 | rs9767451 | T | C | GENO | 30/374/878 | 95/926/1947 | 4.58 | 0.1013 |
|  |  |  |  | ALLELIC | 434/2130 | 1116/4820 | 4.22 | 0.0400 |
| 6 | rs72967259 | T | C | GENO | 19/226/1037 | 32/499/2437 | 1.73 | 0.4211 |
|  |  |  | C | ALLELIC | 264/2300 | 563/5373 | 1.34 | 0.2463 |
| 6 | rs77885036 | T | A | GENO | 18/236/1028 | 35/505/2428 | 1.65 | 0.4384 |
|  |  |  |  | ALLELIC | 272/2292 | 575/5361 | 1.70 | 0.1928 |
| 6 | rs11153693 | C | T | GENO | 181/610/491 | 439/1419/1110 | 0.48 | 0.7866 |
|  |  |  |  | ALLELIC | 972/1592 | 2297/3639 | 0.47 | 0.4939 |
| 6 | rs74296487 | G | A | GENO | 10/167/1105 | 18/374/2576 | 0.57 | 0.7506 |
|  |  |  |  | ALLELIC | 187/2377 | 410/5526 | 0.41 | 0.5224 |
| 6 | rs111583609 | A | G | GENO | 94/526/662 | 211/1211/1546 | 0.11 | 0.9472 |
|  |  |  |  | ALLELIC | 714/1850 | 1633/4303 | 0.10 | 0.7497 |
| 6 | rs6899715 | A | G | GENO | 136/577/569 | 350/1300/1318 | 1.39 | 0.4983 |
|  |  |  |  | ALLELIC | 849/1715 | 2000/3936 | 0.27 | 0.6029 |
| 6 | rs78845922 | T | C | GENO | 24/308/950 | 44/689/2235 | 1.27 | 0.5293 |
|  |  |  |  | ALLELIC | 356/2208 | 777/5159 | 0.98 | 0.3223 |
| 6 | rs1541317 | G | T | GENO | 103/510/669 | 252/1199/1517 | 0.51 | 0.7758 |
|  |  |  |  | ALLELIC | 716/1848 | 1703/4233 | 0.51 | 0.4736 |
| 16 | rs12922283 | C | G | GENO | 46/359/877 | 92/819/2057 | 0.82 | 0.6649 |
|  |  |  |  | ALLELIC | 451/2113 | 1003/4933 | 0.61 | 0.4363 |
| 16 | rs12596974 | C | A | GENO | 109/500/673 | 246/1193/1529 | 0.54 | 0.7652 |
|  |  |  |  | ALLELIC | 718/1846 | 1685/4251 | 0.13 | 0.7189 |
| 16 | rs117267808 | A | G | GENO | 9/218/1055 | 9/380/2579 | 16.81 | 0.0002 |
|  |  |  |  | ALLELIC | 236/2328 | 398/5538 | 16.21 | 5.68×10^-5^ |
| 16 | rs141536185 | A | G | GENO | 6/167/1109 | 9/326/2633 | 4.41 | 0.1105 |
|  |  |  |  | ALLELIC | 179/2385 | 344/5592 | 4.36 | 0.0367 |
| 16 | rs4430753 | A | G | GENO | 70/418/794 | 119/932/1917 | 5.64 | 0.0598 |
|  |  |  |  | ALLELIC | 558/2006 | 1170/4766 | 4.66 | 0.0309 |
| 16 | rs7185180 | C | T | GENO | 9/185/1088 | 14/416/2538 | 1.03 | 0.5975 |
|  |  |  |  | ALLELIC | 203/2361 | 444/5492 | 0.49 | 0.4851 |
| 16 | rs4411505 | C | T | GENO | 15/303/964 | 48/700/2220 | 1.23 | 0.5413 |
|  |  |  |  | ALLELIC | 333/2231 | 796/5140 | 0.28 | 0.5986 |
| 16 | rs4420538 | T | A | GENO | 16/292/974 | 47/695/2226 | 0.95 | 0.6217 |
|  |  |  |  | ALLELIC | 324/2240 | 789/5147 | 0.68 | 0.4111 |
| 16 | rs11074451 | G | A | GENO | 16/215/1051 | 24/451/2493 | 3.69 | 0.1580 |
|  |  |  |  | ALLELIC | 247/2317 | 499/5437 | 3.37 | 0.0665 |
| 16 | rs4643331 | T | C | GENO | 18/238/1026 | 27/534/2407 | 2.35 | 0.3081 |
|  |  |  |  | ALLELIC | 274/2290 | 588/5348 | 1.20 | 0.2738 |

CHR: chromosome; A1: tested allele (minor allele); A2: major allele; GENO: Genotypic analysis; ALLELIC: allelic analysis; AFF: number of patients; UNAFF: number of controls.

**Supplemental Table S3.** Full results for logistic models fitted for each SNP with age and prepregnancy BMI being adjusted.

| CHR | SNP | POS | A1 | OR | T-Statistics | *P* |
| --- | --- | --- | --- | --- | --- | --- |
| 6 | rs80196932 | 117675468 | C | 0.74 | -4.19 | 2.79×10^-5^ |
| 6 | rs9767451 | 117675655 | T | 0.87 | -2.12 | 0.0341 |
| 6 | rs72967259 | 117676293 | T | 1.09 | 1.15 | 0.2505 |
| 6 | rs77885036 | 117685173 | T | 1.10 | 1.26 | 0.2089 |
| 6 | rs11153693 | 117686129 | C | 0.97 | -0.73 | 0.4676 |
| 6 | rs74296487 | 117686329 | G | 1.06 | 0.62 | 0.5371 |
| 6 | rs111583609 | 117695136 | A | 1.02 | 0.34 | 0.7354 |
| 6 | rs6899715 | 117695611 | A | 0.97 | -0.51 | 0.6068 |
| 6 | rs78845922 | 117699138 | T | 1.07 | 0.96 | 0.3387 |
| 6 | rs1541317 | 117702153 | G | 0.96 | -0.73 | 0.4659 |
| 16 | rs12922283 | 20309986 | C | 1.05 | 0.74 | 0.4607 |
| 16 | rs12596974 | 20310838 | C | 0.98 | -0.35 | 0.7230 |
| 16 | rs117267808 | 20311846 | A | 1.42 | 4.00 | 6.25×10^-5^ |
| 16 | rs141536185 | 20315755 | A | 1.22 | 2.04 | 0.0418 |
| 16 | rs4430753 | 20320878 | A | 1.13 | 2.12 | 0.0342 |
| 16 | rs7185180 | 20321195 | C | 1.06 | 0.69 | 0.4933 |
| 16 | rs4411505 | 20323357 | C | 0.96 | -0.56 | 0.5772 |
| 16 | rs4420538 | 20323485 | T | 0.94 | -0.87 | 0.3850 |
| 16 | rs11074451 | 20324935 | G | 1.15 | 1.77 | 0.0770 |
| 16 | rs4643331 | 20326883 | T | 1.09 | 1.07 | 0.2869 |

CHR: chromosome; POS: position; A1: tested allele (minor allele).

**Supplemental Table S4.** Results of the haplotype based association analyses.

| Locus | Gene | Haplotype | F_A | F_U | χ^2^ | DF | *P* | SNPs |
| --- | --- | --- | --- | --- | --- | --- | --- | --- |
| H1 | *NUS1* | OMNIBUS | - | - | 30.30 | 2 | 2.64×10^-7^ | rs80196932-rs9767451 |
|  |  | CT | 0.11 | 0.15 | 19.90 | 1 | 8.17×10^-6^ |  |
|  |  | TT | 0.06 | 0.04 | 12.98 | 1 | 0.0003 |  |
|  |  | TC | 0.83 | 0.81 | 4.02 | 1 | 0.0449 |  |
| H2 | *NUS1* | OMNIBUS | - | - | 3.14 | 2 | 0.2083 | rs77885036-rs11153693 |
|  |  | TC | 0.10 | 0.10 | 1.48 | 1 | 0.2237 |  |
|  |  | AC | 0.28 | 0.29 | 2.29 | 1 | 0.1301 |  |
|  |  | AT | 0.62 | 0.61 | 0.44 | 1 | 0.5090 |  |
| H3 | *NUS1* | OMNIBUS | - | - | 2.16 | 2 | 0.3405 | rs111583609-rs6899715 |
|  |  | AA | 0.28 | 0.27 | 0.07 | 1 | 0.7960 |  |
|  |  | GA | 0.05 | 0.06 | 2.15 | 1 | 0.1422 |  |
|  |  | GG | 0.67 | 0.66 | 0.24 | 1 | 0.6214 |  |
| H4 | *NUS1* | OMNIBUS | - | - | 3.43 | 2 | 0.1801 | rs78845922-rs1541317 |
|  |  | TG | 0.14 | 0.13 | 0.84 | 1 | 0.3600 |  |
|  |  | CG | 0.14 | 0.16 | 3.02 | 1 | 0.0821 |  |
|  |  | CT | 0.72 | 0.71 | 0.49 | 1 | 0.4853 |  |
| H5 | *GP2* | OMNIBUS | - | - | 1.78 | 2 | 0.4108 | rs12922283-rs12596974 |
|  |  | CC | 0.17 | 0.17 | 0.44 | 1 | 0.5084 |  |
|  |  | GC | 0.11 | 0.12 | 1.56 | 1 | 0.2113 |  |
|  |  | GA | 0.72 | 0.72 | 0.11 | 1 | 0.7443 |  |
| H6 | *GP2* | OMNIBUS | - | - | 29.33 | 3 | 1.91×10^-6^ | rs117267808-rs141536185-rs4430753 |
|  |  | AAA | 0.07 | 0.06 | 3.61 | 1 | 0.0573 |  |
|  |  | AGA | 0.02 | 0.01 | 25.13 | 1 | 5.35×10^-7^ |  |
|  |  | GGA | 0.13 | 0.13 | 0.17 | 1 | 0.6822 |  |
|  |  | GGG | 0.78 | 0.80 | 4.84 | 1 | 0.0278 |  |

F_A: haplotype frequency in patients; F_U: haplotype frequency in controls; DF: degree of freedom.

**Supplemental Table S5.** eQTL signals of SNP rs80196932 on gene *NUS1* in multiple types of human tissues.

| Gene | SNP | *P* | NES | T-statistic | Tissue |
| --- | --- | --- | --- | --- | --- |
| *NUS1* | rs80196932 | 2.11×10^-43^ | 0.63 | 17.00 | Pancreas |
| *NUS1* | rs80196932 | 1.30×10^-11^ | 0.20 | 6.90 | Artery - Tibial |
| *NUS1* | rs80196932 | 1.80×10^-10^ | 0.30 | 6.60 | Stomach |
| *NUS1* | rs80196932 | 3.50×10^-10^ | 0.28 | 6.50 | Testis |
| *NUS1* | rs80196932 | 5.10×10^-7^ | 0.15 | 5.10 | Muscle - Skeletal |
| *NUS1* | rs80196932 | 7.30×10^-7^ | 0.19 | 5.10 | Breast - Mammary Tissue |
| *NUS1* | rs80196932 | 1.00×10^-6^ | 0.16 | 5.00 | Artery - Aorta |
| *NUS1* | rs80196932 | 2.10×10^-6^ | 0.30 | 4.90 | Brain - Cerebellum |
| *NUS1* | rs80196932 | 3.90×10^-6^ | 0.28 | 4.80 | Pituitary |
| *NUS1* | rs80196932 | 1.80×10^-5^ | 0.27 | 4.40 | Prostate |
| *NUS1* | rs80196932 | 2.90×10^-5^ | 0.13 | 4.20 | Lung |
| *NUS1* | rs80196932 | 3.10×10^-5^ | 0.13 | 4.20 | Skin - Sun Exposed (Lower leg) |
| *NUS1* | rs80196932 | 0.0001 | 0.15 | 3.90 | Heart - Left Ventricle |
| *NUS1* | rs80196932 | 0.0002 | 0.18 | 3.90 | Artery - Coronary |
| *NUS1* | rs80196932 | 0.0002 | 0.12 | 3.80 | Esophagus - Muscularis |
| *NUS1* | rs80196932 | 0.0015 | 0.24 | 3.20 | Brain - Cerebellar Hemisphere |
| *NUS1* | rs80196932 | 0.0017 | 0.10 | 3.20 | Nerve - Tibial |
| *NUS1* | rs80196932 | 0.0017 | 0.14 | 3.20 | Spleen |
| *NUS1* | rs80196932 | 0.0025 | 0.09 | 3.00 | Adipose - Subcutaneous |
| *NUS1* | rs80196932 | 0.0033 | 0.18 | 3.00 | Small Intestine - Terminal Ileum |
| *NUS1* | rs80196932 | 0.0036 | 0.18 | 3.00 | Brain - Nucleus accumbens (basal ganglia) |
| *NUS1* | rs80196932 | 0.0041 | 0.09 | 2.90 | Adipose - Visceral (Omentum) |
| *NUS1* | rs80196932 | 0.0056 | 0.11 | 2.80 | Colon - Transverse |
| *NUS1* | rs80196932 | 0.0063 | 0.07 | 2.70 | Whole Blood |
| *NUS1* | rs80196932 | 0.0140 | 0.14 | 2.50 | Minor Salivary Gland |
| *NUS1* | rs80196932 | 0.0170 | 0.18 | 2.40 | Brain - Putamen (basal ganglia) |
| *NUS1* | rs80196932 | 0.0190 | 0.11 | 2.40 | Liver |
| *NUS1* | rs80196932 | 0.0200 | -0.05 | -2.30 | Cells - Cultured fibroblasts |
| *NUS1* | rs80196932 | 0.0200 | 0.26 | 2.40 | Cells - EBV-transformed lymphocytes |
| *NUS1* | rs80196932 | 0.0230 | 0.17 | 2.30 | Brain - Anterior cingulate cortex (BA24) |
| *NUS1* | rs80196932 | 0.0250 | 0.19 | 2.30 | Ovary |
| *NUS1* | rs80196932 | 0.0260 | 0.19 | 2.30 | Uterus |
| *NUS1* | rs80196932 | 0.0320 | 0.06 | 2.10 | Skin - Not Sun Exposed (Suprapubic) |
| *NUS1* | rs80196932 | 0.0340 | 0.10 | 2.10 | Colon - Sigmoid |
| *NUS1* | rs80196932 | 0.0890 | 0.07 | 1.70 | Heart - Atrial Appendage |
| *NUS1* | rs80196932 | 0.1500 | -0.04 | -1.40 | Esophagus - Mucosa |
| *NUS1* | rs80196932 | 0.1500 | -0.04 | -1.40 | Thyroid |
| *NUS1* | rs80196932 | 0.2300 | 0.06 | 1.20 | Adrenal Gland |
| *NUS1* | rs80196932 | 0.3000 | 0.10 | 1.00 | Vagina |
| *NUS1* | rs80196932 | 0.4700 | 0.04 | 0.72 | Brain - Cortex |
| *NUS1* | rs80196932 | 0.5200 | 0.07 | 0.64 | Brain - Spinal cord (cervical c-1) |
| *NUS1* | rs80196932 | 0.6200 | 0.03 | 0.50 | Brain - Frontal Cortex (BA9) |
| *NUS1* | rs80196932 | 0.6900 | 0.04 | 0.40 | Brain - Substantia nigra |
| *NUS1* | rs80196932 | 0.8000 | 0.01 | 0.25 | Brain - Hypothalamus |
| *NUS1* | rs80196932 | 0.8200 | -0.02 | -0.22 | Brain - Caudate (basal ganglia) |
| *NUS1* | rs80196932 | 0.9400 | -0.01 | -0.08 | Brain - Hippocampus |
| *NUS1* | rs80196932 | 0.9500 | 0.01 | 0.07 | Brain - Amygdala |

NES: normalized effect size.

Threshold of *P* values was 0.05/47≈0.001.

**Supplemental Table S6.** eQTL signals of SNP rs117267808 on gene *GP2* in multiple types of human tissues.

| Gene | SNP | *P* | NES | T-statistic | Tissue |
| --- | --- | --- | --- | --- | --- |
| *GP2* | rs117267808 | 0.01 | -1.30 | -2.70 | Ovary |
| *GP2* | rs117267808 | 0.07 | 0.82 | 1.80 | Adrenal Gland |
| *GP2* | rs117267808 | 0.09 | -0.57 | -1.70 | Brain - Hypothalamus |
| *GP2* | rs117267808 | 0.10 | 0.40 | 1.70 | Heart - Atrial Appendage |
| *GP2* | rs117267808 | 0.13 | 0.29 | 1.50 | Muscle - Skeletal |
| *GP2* | rs117267808 | 0.13 | 0.30 | 1.50 | Skin - Sun Exposed (Lower leg) |
| *GP2* | rs117267808 | 0.16 | 0.35 | 1.40 | Stomach |
| *GP2* | rs117267808 | 0.17 | -0.32 | -1.40 | Esophagus - Muscularis |
| *GP2* | rs117267808 | 0.19 | -0.41 | -1.30 | Pituitary |
| *GP2* | rs117267808 | 0.20 | 0.43 | 1.30 | Brain - Spinal cord (cervical c-1) |
| *GP2* | rs117267808 | 0.20 | -0.18 | -1.30 | Cells - Cultured fibroblasts |
| *GP2* | rs117267808 | 0.22 | -0.46 | -1.20 | Cells - EBV-transformed lymphocytes |
| *GP2* | rs117267808 | 0.23 | -0.50 | -1.20 | Liver |
| *GP2* | rs117267808 | 0.25 | 0.29 | 1.10 | Heart - Left Ventricle |
| *GP2* | rs117267808 | 0.26 | -0.46 | -1.10 | Brain - Hippocampus |
| *GP2* | rs117267808 | 0.27 | 0.41 | 1.10 | Brain - Anterior cingulate cortex (BA24) |
| *GP2* | rs117267808 | 0.28 | 0.26 | 1.10 | Adipose - Visceral (Omentum) |
| *GP2* | rs117267808 | 0.28 | -0.28 | -1.10 | Esophagus - Mucosa |
| *GP2* | rs117267808 | 0.29 | -0.36 | -1.10 | Brain - Cerebellar Hemisphere |
| *GP2* | rs117267808 | 0.37 | -0.19 | -0.91 | Artery - Aorta |
| *GP2* | rs117267808 | 0.42 | -0.26 | -0.81 | Colon - Sigmoid |
| *GP2* | rs117267808 | 0.45 | -0.18 | -0.76 | Lung |
| *GP2* | rs117267808 | 0.47 | 0.16 | 0.73 | Thyroid |
| *GP2* | rs117267808 | 0.53 | -0.18 | -0.63 | Testis |
| *GP2* | rs117267808 | 0.55 | -0.09 | -0.59 | Whole Blood |
| *GP2* | rs117267808 | 0.56 | 0.18 | 0.58 | Brain - Caudate (basal ganglia) |
| *GP2* | rs117267808 | 0.57 | 0.20 | 0.56 | Minor Salivary Gland |
| *GP2* | rs117267808 | 0.58 | 0.20 | 0.56 | Brain - Putamen (basal ganglia) |
| *GP2* | rs117267808 | 0.59 | -0.12 | -0.55 | Adipose - Subcutaneous |
| *GP2* | rs117267808 | 0.63 | -0.12 | -0.48 | Prostate |
| *GP2* | rs117267808 | 0.65 | 0.18 | 0.46 | Brain - Amygdala |
| *GP2* | rs117267808 | 0.65 | 0.10 | 0.45 | Nerve - Tibial |
| *GP2* | rs117267808 | 0.66 | -0.16 | -0.44 | Brain - Substantia nigra |
| *GP2* | rs117267808 | 0.68 | 0.13 | 0.41 | Brain - Cortex |
| *GP2* | rs117267808 | 0.73 | -0.13 | -0.35 | Brain - Frontal Cortex (BA9) |
| *GP2* | rs117267808 | 0.73 | -0.24 | -0.34 | Uterus |
| *GP2* | rs117267808 | 0.75 | -0.08 | -0.32 | Breast - Mammary Tissue |
| *GP2* | rs117267808 | 0.80 | -0.11 | -0.25 | Artery - Coronary |
| *GP2* | rs117267808 | 0.80 | -0.03 | -0.26 | Pancreas |
| *GP2* | rs117267808 | 0.81 | -0.07 | -0.23 | Brain - Cerebellum |
| *GP2* | rs117267808 | 0.81 | -0.10 | -0.25 | Spleen |
| *GP2* | rs117267808 | 0.83 | 0.05 | 0.22 | Skin - Not Sun Exposed (Suprapubic) |
| *GP2* | rs117267808 | 0.86 | -0.06 | -0.18 | Brain - Nucleus accumbens (basal ganglia) |
| *GP2* | rs117267808 | 0.86 | -0.07 | -0.18 | Small Intestine - Terminal Ileum |
| *GP2* | rs117267808 | 0.91 | -0.05 | -0.12 | Vagina |
| *GP2* | rs117267808 | 0.94 | 0.02 | 0.07 | Colon - Transverse |
| *GP2* | rs117267808 | 0.98 | 0.00 | -0.02 | Artery - Tibial |

NES: normalized effect size.

Threshold of *P* values was 0.05/47≈0.001.

**Supplemental Table S7.** Serum level of NUS1 and GP2 in different genotype groups of SNP rs80196932 and rs117267808.

| Serum level of Proteins | SNPs | Genotypes | Disease Status | | | *F*-Statistics | *P* | **P*_adjusted_ |
| --- | --- | --- | --- | --- | --- | --- | --- | --- |
|  |  |  | Cases (N=1,282) | Controls (N=2,968) | Combined (N=4,250) |  |  |  |
| **Serum level of NUS1,ng/ml | rs80196932 | CC | 2.34±1.83 | 1.40±0.49 | 1.58±0.96 |  |  |  |
|  |  | CT | 2.62±2.25 | 1.76±0.74 | 1.98±1.36 |  |  |  |
|  |  | TT | 2.82±2.30 | 2.04±0.86 | 2.29±1.52 | 47.02 | 8.02×10^-12^ | 1.72×10^-12^ |
| Serum level of GP2,pmol/l | rs117267808 | AA | 3.02±1.70 | 2.10±1.03 | 2.56±1.44 |  |  |  |
|  |  | AG | 2.63±2.10 | 1.79±1.09 | 2.10±1.58 |  |  |  |
|  |  | GG | 2.31±1.93 | 1.33±0.90 | 1.61±1.36 | 68.29 | <2×10^-16^ | <2×10^-16^ |

*ANCOVA model adjusted by disease status.

**Serum level of proteins were presented in mean ± standard deviation.


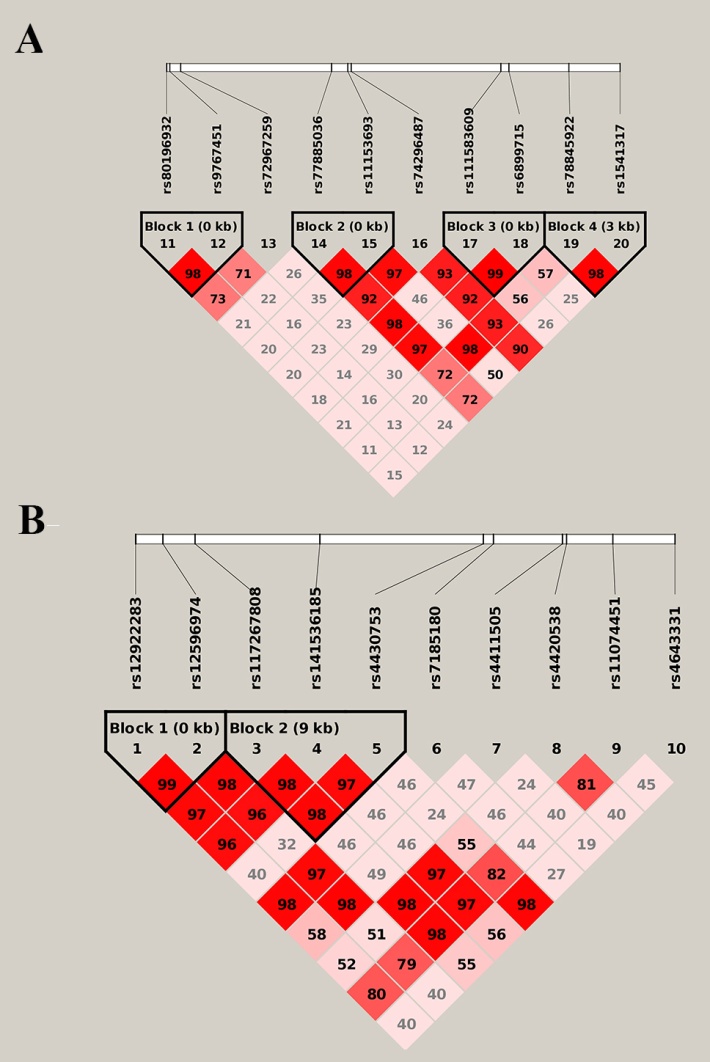


**Supplemental Figure S1.** Linkage disequilibrium plots for genetic polymorphisms genotyped in gene *NUS1* and *GP2*. Values of D' are indicated in each cell. Linkage disequilibrium blocks are indicated in bold lines. A. Linkage disequilibrium plot for genetic polymorphisms genotyped in gene *NUS1*. B. Linkage disequilibrium plot for genetic polymorphisms genotyped in gene *GP2*.


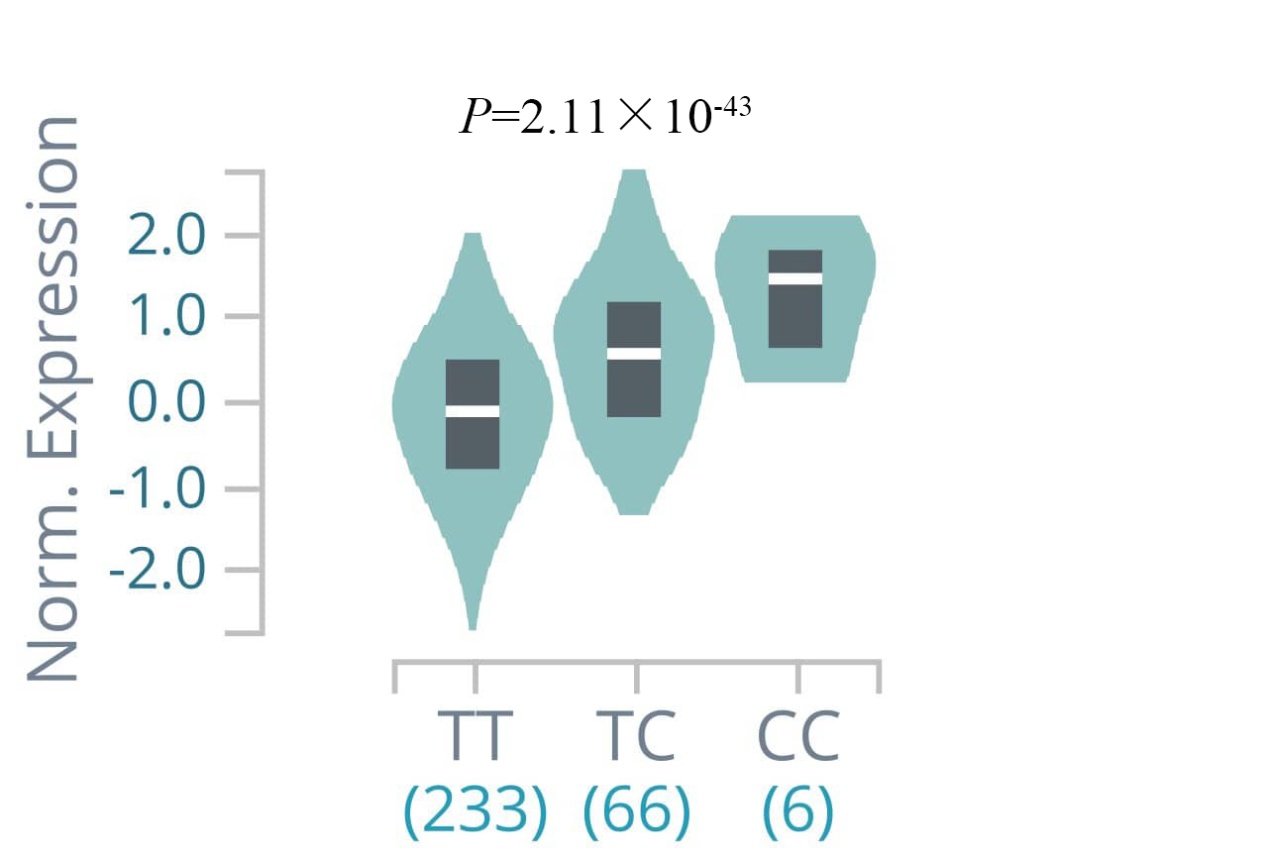


**Supplemental Figure S2.** Normalized gene expression levels of *NUS1* for individuals with different genotypes of SNP rs80196932 in pancreas tissues.
